# Protocol for a multicenter-cluster randomized clinical trial of a motor skills intervention to promote physical activity and health in children: the CHAMP afterschool program study

**DOI:** 10.1186/s12889-022-13849-8

**Published:** 2022-08-13

**Authors:** Leah E. Robinson, Kara K. Palmer, María Enid Santiago-Rodríguez, Nicholas D. Myers, Lu Wang, Karin A. Pfeiffer

**Affiliations:** 1 School of Kinesiology, University of Michigan, Ann Arbor, MI 48109 USA; 2 Department of Kinesiology, College of Education, Michigan State University, East Lansing, MI 48824 USA; 3 School of Public Health, University of Michigan, Ann Arbor, MI 48109 USA

**Keywords:** Children, Youth, Motor competence, Fitness, Moderate to vigorous physical activity

## Abstract

**Background:**

Promoting health-enhancing and sustainable physical activity levels across childhood and adolescence contribute to positive health outcomes as an adult. This study will aim to: a) examine the immediate (pre- to post-intervention) and sustained (1-year post-intervention follow-up) effects of the Children’s Health Activity Motor Program-Afterschool Program (CHAMP-ASP) on physical activity, motor competence, and perceived motor competence relative to the comparison ASP, b) examine the immediate and sustained effects of CHAMP-ASP on secondary health outcomes, specifically health-related physical fitness (i.e., cardiorespiratory fitness, muscular strength, percent body fat) and weight status compared to children in the comparison ASP, and c) determine if perceived motor competence mediates the effect of CHAMP-ASP on moderate-to-vigorous physical activity.

**Methods:**

This multicenter cluster randomized trial will be implemented by ASP staff and will be conducted in ASPs located in two city-based cohorts: East Lansing/Lansing and Ann Arbor/Ypsilanti, Michigan. Children (*N* = 264) who are K-2 graders will participate 35 min/day X 3 days/week for 19 weeks (1995 min) in their afterschool movement program (i.e., CHAMP-ASP vs. comparison). The research team will train ASP staff to implement the program, which will be delivered within the existing ASP offering. Measures of physical activity (accelerometer), motor competence (process and product measures of fundamental motor skills), health-related fitness, perceived motor competence, and anthropometry will be collected pre-, immediately post-, and one-year post-intervention. Random-effects models will be used to assess the clustered longitudinal effect of the intervention on outcome measures.

**Discussion:**

The long-term goal is to provide a sustainable, ecologically-relevant, and evidence-based program during the early elementary years that can be delivered by ASP staff, is health-enhancing, and increases physical activity in children. Findings hold the potential to help shape public health and educational policies and interventions that support healthy development and active living during the early years.

**Trial registration:**

ClinicalTrials.gov Identifier NCT05342701.

**Ethics and dissemination:**

Ethical approval was obtained through the Health Sciences and Behavioral Sciences IRB, University of Michigan (HUM00208311). The CHAMP-ASP study is funded by the National Institutes of Health. Findings will be disseminated via print, online media, dissemination events, and practitioner and/or research journals.

## Background

A consensus report in Pediatrics states that physical activity (PA) is foundational to our health and well-being [1]. Specifically, PA is essential to children’s health (i.e., cardiovascular, musculoskeletal, mental and behavioral, physical, etc) [2, 3]. Additionally, low PA levels are correlated with childhood obesity [2, 3]. Despite the health benefits of PA, data support that 76% of American children and adolescents do not meet physical activity guidelines of 60 min of daily moderate to vigorous PA (MVPA) or equivalent [4, 5]. Thus, research supports the importance of MVPA and the need to support PA in children and adolescents.

Despite this critical need, a knowledge gap in the PA intervention literature related to motor skills is present. Motor skills such as locomotor skills (e.g., running, galloping, and jumping) and ball skills (e.g., dribbling, catching, and throwing) are considered the building blocks that contribute to the development of more complex movement skills required for physical activity engagement [6–8]. Although the literature suggests that it is critical to teach children motor skills at an early age (ages 3–8 years) [6, 9], the instruction of motor skills is not typical in PA interventions. Cross-sectional studies confirm that children who exhibit better competence in their motor skills participate in more health-enhancing PA across childhood and have a healthier weight status [8]. However, relationships between MVPA and motor skills are not fully understood. Thus, more investigations are needed to explore the effect of movement-based instruction to maximize the developmental trajectory of motor skills to support PA and health.

Afterschool programs (ASPs) serve over 10 million children annually [10] and provide an ideal setting for implementing programs designed to increase children’s PA. ASPs allow children to learn, practice, and develop essential skills that many children do not have the opportunity to learn. However, to date, afterschool interventions that address motor skill performance and health-related outcomes in PA have been lacking in the scientific literature. There is a need for interventions that address these movement behaviors in settings commonly attended by youth (e.g., afterschool programs). Moreover, these interventions should be designed to be sustainable.

Motor skill interventions have shown significant improvements in motor skill competence in children aged 3 - 10 years [11–13]. One evidence-based motor skill intervention that has been shown to promote motor skills and PA in young children is the Children’s Health Activity Motor Program (CHAMP). CHAMP is a theoretically-grounded, mastery-based motor skills intervention that enhances motor skills [14–21], PA [19, 22–24], perceived physical competence [15, 25, 26], and constructs of self-regulation [16] (more CHAMP details and specifics are in the methods). Most CHAMP studies have targeted preschool-age children, and the intervention has only been delivered by research staff and personnel with content expertise in motor development. Although the findings support that CHAMP is efficacious, there are concerns regarding the sustainability of CHAMP due to the instruction and implementation of the intervention being research personnel. CHAMP must be empirically tested to verify if it will work when implemented by non-motor development experts (i.e., ASP staff).

The proposed clinical trial aims to test the effects of CHAMP when non-motor development experts implement on motor competence, PA, and health outcomes in children. This study will expand on previous CHAMP work and addresses the following aims and hypotheses.

- Aim 1. To examine the immediate and sustained effects of CHAMP-ASP on MVPA, motor competence, and perceived motor competence (primary outcomes) relative to children randomized to the comparison ASP.
  - Hypothesis 1a. Children in CHAMP-ASP will exhibit more MVPA, more advanced motor competence, and higher perceived motor competence than children in the comparison ASP immediately post-intervention.
  - Hypothesis 1b. Children in CHAMP-ASP will exhibit more MVPA, more advanced motor competence, and higher perceived motor competence compared to children in the comparison ASP at 1-year (post-intervention) follow-up.
- Aim 2. To examine the immediate and sustained effects of CHAMP-ASP on health-related physical fitness (cardiorespiratory fitness, muscular strength; secondary outcomes) and weight status (secondary outcome) relative to children randomized to the comparison ASP.
  - Hypothesis 2a. Children in CHAMP-ASP will demonstrate higher health-related physical fitness levels and better maintenance of BMI z-score compared to children in the comparison ASP immediately post-intervention.
  - Hypothesis 2b*.* Children in CHAMP-ASP will demonstrate higher health-related physical fitness levels and better maintenance of BMI z-score compared to children in the comparison ASP at 1-year (post-intervention) follow-up.
- Exploratory Aim: To determine if perceived motor competence mediates the effect of CHAMP-ASP on PA.

## Methods

### Design

The proposed study is a two-arm, cluster-randomized multicenter trial with 1-year follow-up. ASPs will be randomly assigned to treatment (i.e., CHAMP-ASP) or comparison (i.e., standard of practice). Intervention and comparison ASPs will be matched before randomization based on demographic characteristics (racial/ethnic distribution, percent of children eligible to receive free/reduced-price lunch, etc.) and program type (e.g., provide tutoring or academic content). Matching by program type reduces the risk of differential effects based on the presence/absence of PA programming. This study will take place in 12 ASPs located in central Michigan (*n* = 6 from Ypsilanti/Ann Arbor and *n* = 6 from East Lansing/Lansing). Half of the sites from each city will be randomized to intervention (CHAMP-ASP) and half to comparison.

### Setting, participants, and recruitment

#### Participants

Participants will be at least 264 children, 5–8 years of age (~ 132 per condition; CHAMP-ASP and comparison).

#### Inclusion/exclusion criteria

ASP: This study is open to ASPs willing to accept an assignment to treatment or comparison and have space to conduct motor skill training. If an ASP implements general PA programming, it will not be excluded from participating in this project. In all cases, CHAMP-ASP will be considered a supplemental portion of an existing program. Specifically, ASPs with current movement and PA programs will either serve as a comparison or replace their existing program with CHAMP-ASP. Participants: Children in grades K-2 (~ 5–8 years of age) and present for participation in the ASP for at least 1 h afterschool will be invited to participate in this study. To be enrolled in this study, children must be able to participate in physical education classes (i.e., able to participate in movement and PA), cannot be diagnosed with syndromes, developmental/physical disability, physical disability and/or diseases that affect PA participation, and must be able to understand English. Children who fail to meet these inclusion criteria will not be enrolled in the study, and data will not be collected on these individuals. However, they will still be able to participate in the intervention if allowed by ASP staff.

#### ASP recruitment

We will recruit from existing relationships with schools and ASP providers and contact new schools and providers if necessary.

#### ASP staff training

ASP staff will be trained to instruct CHAMP with a *‘train the trainer’* approach due to the opportunity to learn by teaching and its potential for better retention [27]. Staff training will occur over approximately 4 weeks. During the first 2 weeks, ASP staff will have access to the CHAMP-ASP staff online training and complete five online learning modules. The online modules focus on an overview of 1) motor skills and other health-related outcomes in young children, 2) understanding of the CHAMP program, including underlying theory and TARGET structures, 3) how to create and implement the CHAMP in the ASP, and 4) an overview of the CHAMP curriculum. Each module will have embedded, pre-recorded videos (*n* = 1–4 per module) and written text. After each pre-recorded video, ASP staff will complete online learning checks to examine their understanding of the materials. After completing the online training portion, ASP staff will begin the in-person CHAMP training across a two-week period. This training will implement CHAMP sessions which are scaffolded to fade out assistance from the research staff.

#### Formative assessment

Since this study represents the first time individuals other than research staff will deliver the intervention, it was essential to test the training materials and select them in a feasibility study. Before the initiation of the main study, ASP staff from two sites will undergo staff training as outlined in the ASP Staff training. Using a scaffolding approach, these staff members will also teach a series of CHAMP lessons (i.e., co-teaching with research staff leading to independent instruction). The first series of lessons will be conducted with the research staff so ASP staff can participate and watch as necessary. A second set will be completed entirely by ASP staff with minimal assistance from research staff, and the third series of lessons will be fully conducted by ASP staff with research staff evaluating their instruction. In addition to evaluation by research staff, semi-structured interviews will be conducted after the third session to provide feedback to improve CHAMP training. We will publish full details regarding the design and implementation of the training module for ASP staff, and the findings will be published separately as it is not an aim of this project.

#### CHAMP intervention and implementation

The CHAMP intervention will consist of a dose of 1995 min total (35 min/day*3 days/week*19 weeks). Each session will consist of three parts: 1) 3–5 min of warm-up & introductory activity (i.e., verbal and visual demonstration of skill/activity and critical elements of each skill introduced at varying levels to meet the developmental needs of the learners); 2) 25 min of motor skill instruction and practice (e.g., children self-navigate as it relates to their choice of activity, level of difficulty, and interaction with peers) via 3–4 stations (minimum of 1 locomotor and 1 ball skill) that have 3–4 levels of difficulty, with instructors providing individualized and specific feedback; and 3) 3–5 min closure activity (i.e., recalling the critical elements of each motor skill for each station). As previously outlined, CHAMP will be implemented in the ASPs by ASP staff who were trained with a ‘train the trainer’ approach. An additional research staff member, a process evaluator, will be present at sessions to complete the process evaluation, including a fidelity check of the intervention. Classroom teachers are not involved in implementing CHAMP-ASP since ASP staff are hired to conduct afterschool programming. However, teachers (physical education and classroom) will be informed about the CHAMP-ASP project occurring in their school.

#### Comparison

The ASP sites offer various options, mainly in the form of clubs with academic activities and homework, arts and crafts, and sometimes unstructured PA. The programs provide unstructured PA and lack formalized instruction and feedback on motor skill. The comparison arm for this study will deliver standard of practice in ASPs and be evaluated with a process evaluation.

#### Process evaluation

Feasibility, acceptability, reach, dose delivered, and fidelity of CHAMP-ASP will be assessed, in both formative and summative formats by the research team with the assistance of the ASP staff. ASP staff will provide evaluations of the training via survey methods for both the online and in-person portions of the training. The feasibility and acceptability of CHAMP will be assessed by surveys and interviews with the ASP staff and child participants, and the data will be used in a summative manner (i.e., reported at the end of the study). Additionally, ASP staff who conduct the intervention will provide feedback regarding how well the program applies to K-2 children and note any modifications they make at the end of each intervention week; this will take place via brief surveys. Reach and dose delivered will be assessed by a research staff member via attendance (i.e., reach), checklists (to note delivery of program components), and recording of minutes of CHAMP intervention. Reach and dose delivered will be assessed summatively. Adverse events and serious adverse events will also be recorded at the treatment site.

ASP staff will wear wireless microphones to assess fidelity during intervention sessions, and CHAMP will be digitally recorded. Fidelity checks will be conducted during the live intervention sessions by research staff and digital recording, using a systematic observation tool established for mastery interventions [25, 28–31]. Fidelity checks will be conducted across the duration of the intervention (e.g., weeks 2, 3, 6, 9, 13, and 15). ASP staff who conduct the intervention will receive feedback regarding their performance within 1 week, and this information will be used in a formative fashion. ASP directors and ASP staff will also be questioned about the afterschool environment during brief interviews (post-intervention); this includes documenting secular changes at comparison sites via interviews. ASP staff who conduct the intervention will be incentivized to provide information. Interviews will be facilitated by a graduate student trained in qualitative research. A trained research assistant will transcribe each session. Content and thematic analysis will be conducted to identify themes generated by qualitative data collection sessions.

### Measures and outcomes

Measurements will be collected at baseline (pre-intervention), immediately following the intervention (post-intervention), and at the 1-year post-intervention follow-up. All measures will be collected at each ASP over a 3–4 week period. Data will be collected by trained, blinded research assistants. The ASP staff will have no role in data collection but will assist with the management of children. Privacy screens will be used to collect sensitive measures, and if the site has other rooms available, data collection will occur in a separate room. The following text provides descriptions of each variable and the operational definition.

#### Demographic variables

Date of birth, biological sex, and race/ethnicity will be collected through self-report during the parental consent process.

#### Physical activity

Time spent in sedentary, light, and MVPA will be examined with ActiGraph GT3X-BT accelerometers for 1 week at each measurement point (baseline, post-intervention, and 1-year post-intervention). Children will wear the devices on the non-dominant wrist on a plastic band and will not remove the devices for water activities or sleep. Accelerometers will be set to collect data in raw mode (30 Hz) and processed using ActiLife software and R code. Cut-points, likely from Hildebrand et al. [32] or Crotti et al. [33], will be used to determine time spent in various intensities (sedentary, light, moderate, vigorous). To be included in analyses, participants will need to achieve 10 hours of wear time on at least 3 weekdays and 1 weekend day [34]. Compliance will be enhanced by the wrist placement because participants will not need to remove the monitor during the measurement period.

#### Motor competence

Motor competence will be measured with process and product measures of motor skills which will be examined concurrently in this study. Process measures will be assessed using the Test of Gross Motor Development (TGMD, 3rd edition), a validated assessment of motor competence in children 3–10 years old [35]. Sum of locomotor and ball skills scores (0–100) will be used to compute total score. Completing both locomotor and ball skills measures of motor competence increases the predictive validity of PA. Additionally, separate scores for the locomotor and ball skills subscales will be examined. Locomotor score is the summed scores of process characteristics exhibited for each of 6 locomotor skills (run, jump, gallop, slide, hop, and skip; 0–46). Ball skills score is the summed score of process characteristics exhibited for each of 7 ball skills (overhand throw, catch, dribble, underhand throw, kick, one-handed strike, two-handed strike; 0–54). Mean test-retest reliability coefficients were reported as 0.88 for locomotor and 0.93 for ball skills items with interrater reliability coefficients for both subscales of 0.98 [35]. Assessments will be digitally recorded and coded by an individual blind to the randomization. Inter-rater reliability will be established between the blind coder and two members of the project. Each member will establish reliability on 25% of the data between themselves and the blind coder and on 10% of the data among all three coders to ensure consistency.

Product measures of motor skills will be assessed according to established protocol and procedures [36–39]. Ball (throw) speed (m/s) of a tennis ball will be measured with a radar gun [35, 37–41]. The max speed of five throws will be used in analyses. Kick speed (m/s) will be measured with a radar gun [36, 38–42]. The child will be instructed to approach the ball and kick the ball as hard as they can toward the wall. The max speed (m/s) of five kicks will be used in analyses. Running speed (m/s) will be assessed with a radar gun based on time to run 18.3 m. Three run trials will be recorded and the max speed will be used in analyses. Standing long jump distance (cm) will be measured across five trials with the max of all five jump attempts used for analyses [36, 38–40]. Hop speed will be measured using video analyses [36, 38]. Each child will be instructed to hop on 1 ft consecutively four times on each foot as fast as they can. The research assistant will record the time taken to complete four hops on each foot for two trials. The average of the max hop speed across both feet will be used in the analyses. If a child is unable to hop four times, they will receive a score of 0 for that trial. Product scores will be standardized and summed across ball skills (throw and kick) and locomotor skills (run, jump, hop) and across all five skills. Summed standardized scores will be used in analyses [36–39].

#### Perceived motor competence

Perceived motor competence will be assessed with two instruments, Harter and Pike’s Pictorial Scale of Perceived Competence and Social Acceptance for Young Children (PSPCSA), which measures global perceived physical competence, and the Digital Scale of Perceived Motor Competence (DSPMC), which measures perceived motor competence [43, 44]. The average of 6 items on PSPCSA scale (0–4) will be used for analysis. Reliability (internal consistency) of the individual items ranged from alpha of 0.65–0.89, with a reliability ICC = 0.89 for the total scale [44]. The DSPMC is a 12-item video-based assessment that examines children’s perceived motor competence [45, 46]. Reliability (internal consistency) on locomotor vs ball skill subscale ranged from alpha of 0.69 to 0.84, with the reliability of ICC = 0.84 for the combined subscale measure [45, 46]. The average of 12 items on DSPMC scale, 0–4 will be used for data analysis. The PSPCSA and DSPMC have been tested and validated in early childhood populations. For both assessments, children will (1) select the picture/video that is most like them (one picture/video depicts a child who is competent, and the other shows an unskilled child); and (2) focus on the designated pictures/videos and indicate whether they are just a “little bit” or “a lot” like that child. Separate pictures/videos for girls and boys will be used. The range of scores for each item on the subscale is 1 (low competence) to 4 (high competence).

#### Cardiorespiratory endurance, muscular strength, and body composition

Cardiorespiratory endurance, muscular strength, and body composition will be used to assess health-related fitness. Cardiorespiratory endurance will be assessed with the 6-min walk test. Participants will engage in one trial where they walk around two cones, placed 30 m apart, as fast as possible. They will be instructed not to run or compete during the trial. Research assistants will provide a warning with 1 min left and record the number of laps completed (which will eventually be converted to distance in meters). Research assistants will mark the spot where the participant finishes on the final lap so that distance can be measured [47]. Upper body muscular strength will be assessed using handgrip strength with a dynamometer, which has been deemed reliable and valid in 6–12-year-olds [48–50]. Grip will be appropriately adjusted for size, and two trials on the right and left sides (elbow extended) will be performed. The sum of maximum score (kg) of two trials for each hand and used in analyses. Body composition (percent body fat) will be assessed via bioelectrical impedance analysis, which has been deemed reliable [51, 52] and valid [51]. The average of two measurements assessed (to the nearest 0.1%, respectively) with a bioelectric impedance analysis scale will be used to collect percent body fat (TANITA Body Composition Scale - SC-331S, Arlington Heights, IL).

#### Height and weight

Height and weight will be used to calculate body mass index (BMI) as an assessment of weight status. Both height and weight will be assessed with the participant barefoot and in light clothing to calculate BMI. Standing height will be measured using a portable stadiometer (Shorr Productions, Olney, MD), and the average of two measurements to the nearest 0.1 cm will be used. BMI will be calculated using age and sex and the CDC growth charts [53], transformed into BMI z-score for analyses. Inter- and intra-rater reliability of data collection staff will be assessed pre-data collection and monitored throughout data collection and must be at 80% agreement in order to be cleared for participation in data collection. For quality control assessment during data collection, every 15th child will be re-measured by a second data collector. If measures are different by > 1 cm, 0.5 kg, and 0.5% body fat, the measurement team will be re-trained.

### Data collection protocol and procedures

Trained research staff not involved in intervention delivery will collect data using standardized instruments. In the first months of the academic year, informed consent will be obtained and will be followed by the collection of pretest measures. The plan is for data collection to occur across three, 20–40-min sessions during the regular ASP times. Session 1 will assess height, weight, body composition, perceived motor competence, and health-related fitness measures. Sessions 2 and 3 will be used to assess motor competence measures in small groups of 4–5 children. PA will be collected between Sessions 1 and 2 (participants will be equipped with an accelerometer at the end of Session 1 and return it at Session 2; see Physical Activity section for further details). Incentives to support recruitment efforts will include: a) Parent/guardian upon returning the IRB consent letter, will be compensated for their time and will receive a one-time $5.00 cash incentive, regardless of consent to participate in the study. b) Children (treatment and comparison) will receive a cash incentive of $10.00 upon the return of the accelerometer at each time point (i.e., baseline, post-intervention, and follow-up; totaling a maximum of $30.00 across the study period), c) Completed Family Questionnaires will be entered into a drawing to win 1 of 8, $25.00 cash drawings, and d) ASP Staff who served as instructors for the project will be compensated for the time and work ($1320.00). We will provide additional incentives to children for their participation (e.g., pencils, stickers, wristbands). See Table 1 for a full timeline of data collection.

**Table 1:** Standard Protocol Items: Recommendations for Interventional Trials diagram for the schedule of enrollment, interventions, and assessments

	Oct 2022	Nov 2022 - April 2023	May 2023	May 2024
Enrollment	X^1^; *n* = 132 X^2^; *n* = 132			
Intervention				
CHAMP		X^1^; *n* = 66 X^2^; *n* = 66		
Standard of Practice		X^1^; *n* = 66 X^2^; *n* = 66		
Assessment				
Baseline	X^1^; X^2^			
Post-intervention			X^1^; X^2^	
Follow Up				X^1^; X^2^

X^1^ = Site 1, Ann Arbor/Ypsilanti; X^2^ = Site 2, East Lansing/Lansing. See detailed description of collected variables in main protocol. Demographics will be evaluated through parental report. PA will be measured for one full week (ie, 5 weekdays and 2 weekend days) with accelerometers. Motor performance will be evaluated using the Test of Gross Motor Development-third edition (process measures) and product measures of motor skills. Perceived Motor Competence will be assessed with the Pictorial Scale of Competence and Social Acceptance (physical subtest only) and the Digital-Scale of Perceived Motor Competence. CHAMP, Children’s Health Activity Motor Program

#### Training of data collection staff

The data collection staff will be trained in all assessments prior to the onset of data collection by the investigators and research team. The intra- and inter-observer measurement error for anthropometry will be determined. This training protocol has resulted in small measurement errors during the actual measurement period.

### Statistical analyses

#### Analysis of primary and secondary outcomes

We will examine the range and frequency distributions for all variables to detect potential outliers and will transform variables when appropriate. To study both short-term (immediate) and long-term (delayed or sustainable) effects of the CHAMP-ASP intervention, we will assess all longitudinal covariates and outcomes by both summary statistics and descriptive data figures at baseline and post-intervention, and at one-year post-intervention. Since the samples are collected in different ASPs, the data will be clustered, thus we will use Mixed Model Regression, Growth Curve Modeling, and Structural Equation Modeling [54–58] to account for both within-school and within-class correlations, as well as longitudinal correlations, that may exist in the study.

More specifically, the change of motor competence, perceived motor competence, and PA immediately after completing CHAMP-ASP and 1 year after will be compared between two treatment groups using Mixed Model Regression models to examine the immediate and sustained effects of CHAMP-ASP on PA, motor competence, and perceived motor competence (primary outcomes) relative to the comparison ASP, adjusting for other confounding factors such as sex. We will also investigate the amount of attrition from pre- to post-intervention, and 1 year later, and attempt to identify baseline (i.e., pre-intervention) and time-varying predictors of the likelihood of dropping out (as an indication of possible bias in the change estimates). We will specifically test interactions between intervention and time, as well as between baseline motor skill and time to see which baseline measure is a stronger driver of increasing PA. To explore the potential effect modification of sex on the intervention, we will evaluate the interaction term of sex, intervention and time as well. We will also explore the location effect by comparing Ann Arbor/Ypsilanti versus East Lansing/Lansing and include location as a covariate in all models.

Similar statistical analysis methods will be applied to examine the immediate and sustained effects of CHAMP-ASP on secondary health outcomes relative to the extant ASP, with the outcome variable changed to health-related physical fitness (distance, grip strength (kg), percent body fat) and weight status (BMI-z). In these analyses, we will obtain the estimates of the long-term longitudinal intervention effect by adjusting for confounding factors (sex, location, and PA) [54–56, 58]. For weight status, we expect a delay before any significant change is observed because of the difficulty of achieving health behavior change. To address this delay, we will add time-lagged covariates related to behavior changes in the model, as well as the corresponding interaction effects, to understand potential modified intervention effects by different levels of behavior changes.

We will further explore the causal framework and fit counterfactual Growth Curve Models to understand if, and how, time-course changes in motor competence, perceived motor competence, and PA differ causally between the intervention and comparison groups. This model is also used to determine a time window over which the intervention effect appears stronger. We will use Structural Equation Models to determine which hypothesized constructs might be responsible for intervention effect on longitudinal outcomes [55, 56, 58]. Based on the results of our previous work, for example, we hypothesize that the intervention will improve children’s motor competence and enhance children’s perceived motor competence immediately post-intervention, which is then expected to be associated with improved PA when the study has ended. Structural equation models allow us to assess the effects of interpersonal and intrapersonal mediators.

#### Sample size, power analysis and sample attrition

The sample size was calculated based on our primary endpoint, the change in PA between baseline and post-intervention. According to previous literature, we anticipate the changes in the primary endpoint PA for the intervention group after completing the CHAMP-ASP will be higher than that of the comparison group by 12 min of MVPA (whole day), with an estimate of the standard deviation (SD) as 26 [59, 60]. Literature shows that such a change is accepted as a clinically meaningful change in improving PA among lower-elementary-school children [59, 60]. Since we will enroll study subjects from multiple ASPs in Ann Arbor/Ypsilanti and East Lansing/Lansing, we accounted for the intracluster correlations in the following calculations. Based on our previous studies, the intracluster correlation coefficient (ICC) ranges from 0.02 to 0.05. We assumed a conservative ICC as 0.05 in power and sample size considerations. With 90% power at an alpha level of 0.05, we will need a total of 216 subjects (assuming 18 subjects per ASP, 12 ASPs with 6 randomized to be CHAMP-ASP and 6 comparison groups - 108 subjects per intervention arm) to detect the above group difference. To account for 20% loss to follow-up, we will recruit 264 subjects at baseline (assuming 22 students per ASP, 132 subjects per intervention arm). We will take steps to minimize loss to follow-up, and this extra recruitment will also help us to maintain the statistical power at 90%, with unexpected smaller detectable differences in change of PA scores, and for the secondary endpoint analysis [61].

#### Missing data

Assuming data are missing at random, multiple imputation techniques will be used to replace missing data. Missing data will be assumed to be missing at random if no participant demographics or primary outcomes are correlated with missingness. Mixed-effects models allow for partial information to be included for individuals who may drop out before any post-intervention data collection. Sensitivity analysis over a range of missing data mechanisms will be performed as necessary. We expect only sporadic missing data because research staff will be present to supervise data collection and identify potential problems with missed questions, and so forth. Missing values will be multiple imputed using available covariates by sequential imputation. This approach allows optimal use of available data in analysis involving change measures.

#### Early withdrawal/dropouts

We expect a loss to follow-up in both the intervention and comparison groups. A conservative estimate of this attrition rate is 20% during the study period. We plan to implement intent-to-treat analyses. We will make every effort to collect follow-up data at each time point from individuals who have not withdrawn from the study. Our approach has been to sponsor booster sessions as “classroom reunion/birthday parties” to gather follow-up data. This approach has worked well for bringing children together to reduce potential dropout. We will compare baseline demographics, PA and motor competence groups to determine if differential dropout has occurred.

## Discussion

Research supports that PA has many health benefits, but school-age children are not meeting the current physical activity recommendations [4, 5]. We aim to address low levels of PA in children with a movement-based approach that teaches motor skills. Children need to acquire a level of proficiency in motor skills as these behaviors contribute to an active lifestyle and physical activity engagement [7–9]. This study addresses the underlying mechanisms of PA from a developmental perspective. Motor skills are cumulative and not transient like physical activity [6, 9, 62–64], and developing a foundation in motor skills will influence a child’s PA engagement [4, 5]. In this investigation, we aim to use an evidence-based intervention, CHAMP, that provides developmentally appropriate, context-specific movement activities that promote motor competence in young children with the potential immediate and long-term impact on PA.

To our knowledge, no studies have tested the effects of a mastery climate, motor-based intervention on motor competence, PA, and other health-related outcomes (i.e., health-related fitness) in children attending an afterschool program and delivered by non-motor experts. A unique aspect of this study is that ASP staff will be trained to implement the motor skills intervention (i.e., CHAMP). Using ASP staff to implement the intervention aids in the sustainability of the project [27, 65, 66]. Additionally, we apply a rigorous measurement protocol that uses objective instrumentation to evaluate the effects of the intervention on PA, motor competence, perceived motor competence, and health-related physical fitness. Further, and more importantly, this study is one of few to extensively conduct process evaluation to provide thorough documentation of the CHAMP intervention implementation by ASP staff. Data support that 10 million children participate in ASPs each day, and ASPs provide a great opportunity for children to participate in physical activity outside of the school day. The study could help researchers and practitioners by providing better evidence-informed policies and practices for movement programs ASPs.

## Data Availability

The data from this study are not currently available as this study has yet to take place. The dataset will not be in publicly available repositories. These data will be made available upon reasonable request and after signing a data usage agreement with the PIs. Potential users of the data must agree to conditions of use, including but not limited to, restrictions against attempting to identify study participants, reporting responsibilities, and proper acknowledgment of the data resource.

## Abbreviations

ASP: Afterschool program
BMI: Body mass index
CHAMP: Children’s Health Activity Motor Program
DSPMC: Digital-based Scale of Perceived Motor Competence
ICC: Intracluster Correlation Coefficient
PSPCSA: Pictorial Scale of Perceived Competence and Social Acceptance for Young Children
MVPA: Moderate to vigorous physical activity
